# The Danish Neuro-Oncology Registry: establishment, completeness and validity

**DOI:** 10.1186/s13104-016-2233-x

**Published:** 2016-08-30

**Authors:** Steinbjørn Hansen, Jan Nielsen, René J. Laursen, Birthe Krogh Rasmussen, Bente Mertz Nørgård, Kim Oren Gradel, Rikke Guldberg

**Affiliations:** 1 Department of Oncology, Odense University Hospital, 5000 Odense, Denmark; 2 Institute of Clinical Research, Faculty of Health Sciences, University of Southern Denmark, Odense, Denmark; 3 Center for Clinical Epidemiology, Odense University Hospital & Institute of Clinical Research, University of Southern Denmark, Odense, Denmark; 4 Department of Neurosurgery, Aalborg University Hospital, Aalborg, Denmark; 5 Department of Neurology, Nordsjælland Hospital, University of Copenhagen, Hillerød, Denmark

**Keywords:** Brain neoplasms, Glioma, Validity, Completeness, Database research, Clinical quality indicators

## Abstract

**Background:**

The Danish Neuro-Oncology Registry (DNOR) is a nationwide clinical cancer database that has prospectively registered data on patients with gliomas since January 2009. The purpose of this study was to describe the establishment of the DNOR and further to evaluate the database completeness of patient registration and validity of data.

**Methods:**

The completeness of the number of patients registered in the database was evaluated in the study period from January 2009 through December 2014 by comparing cases reported to the DNOR with the Danish National Patient Registry and the Danish Pathology Registry. The data validity of important clinical variables was evaluated by a random sample of 100 patients from the DNOR using the medical records as reference.

**Results:**

A total of 2241 patients were registered in the DNOR by December 2014 with an overall patient completeness of 92 %, which increased during the study period (from 78 % in 2009 to 96 % in 2014). Medical records were available for all patients in the validity analyses. Most variables showed a high agreement proportion (56–100 %), with a fair to good chance-corrected agreement (k = 0.43–1.0).

**Conclusions:**

The completeness of patient registration was very high (92 %) and the validity of the most important patient data was good. The DNOR is a newly established national database, which is a reliable source for future scientific studies and clinical quality assessments among patients with gliomas.

## Background

The Danish Neuro-Oncology Group (DNOG) is the Danish Multidisciplinary Cancer Group for adult patients with primary brain tumors. The DNOG develops guidelines recommending procedures for the diagnostic process and treatment in patients with brain tumor in Denmark. In an extension, a national clinical database was established to monitor the quality of treatment and the changes in the clinical practice. Hence, the Danish Neuro-Oncology Registry (DNOR) was established under the auspices of DNOG and it has prospectively registered nationwide data on patients with gliomas since January 2009 [1].

In 2014, about 1500 newly diagnosed patients with neoplasms in the brain, meninges and cranial nerves were registered in Denmark according to the National Cancer Registry (NCR). These tumors are a heterogeneous entity of many different tumor types [2]. The glioma is the most common tumor type in adult primary brain tumor patients. Additional to the registration in NCR these patients have been registered with clinical data in the DNOR. Patients with glioma have a life threatening disease, which requires prompt handling from the health care providers. This disease has major implications for the patients and their families’ quality of life. Health care costs are high including complicated neurosurgical operations and technically advanced radiotherapy, as well as expensive chemotherapy. For many of the patients there may be a need for rehabilitation or nursing home care. DNOR is thus a quality clinical database of high relevance, and DNOR publishes annual reports with key clinical quality indicators. This enables to compare own results to pre-specified standards and to detect changes in own results from year to year. These results can also be compared to the reported national levels and regional differences can be detected. The discussion of the results from the annual report is an integrated part of the work in DNOG on updating the national guidelines.

The DNOR provides a systematic prospective collection of clinical data, which ensures an important source for optimizing clinical practice and for scientific investigations. However, the scientific utility requires sufficient completeness of patient registration and high data validity. The purpose of this study was to describe the establishment of the DNOR, to evaluate the patient completeness using central health service registries as the reference, and to determine the validity of the most important variables by comparing to the individual medical records.

## Methods

### Establishment

The DNOR was established by the DNOG and is financially supported by the health care authorities through the Danish Clinical Quality Improvement Program. DNOR is organized with a steering committee of specialists in neurosurgery, medical and radiation oncology, neurology, neuroradiology and pathology, as well as representatives from the Competence Centre for Clinical Epidemiology and Biostatistics, South, Competence Centre for Health Quality and Informatics, and from the Region of Southern Denmark housing the database through the Department of Oncology, Odense University Hospital.

**Fig. 1:**
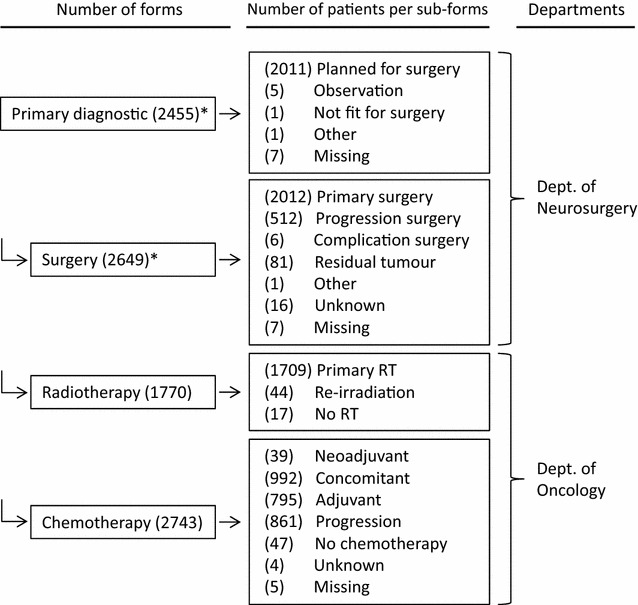
Data model of DNOR with the number of forms entered (*left*) and the distribution of number of patients (*middle*) within each subtype of forms, and departments (*right*) responsible for entering clinical data into DNOR from 2009 to 2014. *Asterisk* some of the patients have been registered with multiple forms

It is mandatory for all departments of neurosurgery and oncology, treating patients with glioma, to report data to the DNOR. Initially, five surgical and six oncological departments participated; this is now reorganized to four neurosurgical and four oncological departments representing the departments treating patients with glioma in Denmark. The data input is web-based through a secured health care network. The data model is organized in four parts (Fig. 1). Each patient has one data form for primary diagnostics, followed by three optional data forms for neurosurgery, radiation therapy, and chemotherapy, all of which can be repeated upon recurrences. The inclusion criteria are patients diagnosed with a primary glioma (not ependymoma) after 1 January 2009 and patients aged ≥18 years at the time of diagnosis.

The data contains information about primary symptoms, characteristics of baseline magnetic resonance imaging (MRI), performance status, operating procedures, the surgeon’s qualification, post-surgical MRI, post-surgical complications, diagnostic classification according to the tenth edition of the International Classification of Diseases (ICD-10), histologic type according the Systematized Nomenclature of Medicine (SNOMED) Morphology (M) codes, and characteristics for radiotherapy and chemotherapy. Eight clinical quality indicators (Table 1) are defined for annual reporting in which department-specific proportions can be compared to the national levels, pre-specified standards, and followed for time trends [3].

**Table 1:** Indicators used for annual reporting in the Danish Neuro-Oncology Registry

No.	Descriptions	Standards (%)
Ia	Proportion of patients who are alive 1 year after surgery out of all patients in whom the histological diagnosis is glioblastoma	≥50
Ib	Proportion of patients who are alive 2 years after surgery out of all patients in whom the histological diagnosis is glioblastoma	≥15
II	Proportion of patients who have undergone postoperative MRI no later than the 3rd day after primary surgery out of all patients who have undergone resection and have contrast-enhancing tumor (i.e., suspected high-grade glioma) prior to resection	≥90
III	Proportion of operations conducted or supervised by a specialist in neurosurgery out of all operations both primary and at progression	≥95
IV	Proportion of patients with no measurable residual tumor demonstrated by postoperative MRI performed after primary surgery out of all primarily diagnosed patients, where the histological diagnosis is glioblastoma. (Denominator includes patients with only performed biopsy or where control MRI scan is not available)	≥20 and ≤70
V	Proportion of patients alive more than 30 days after surgery out of all patients who have undergone primary surgery for gliomas	≥95
VI	Proportion of patients finishing focal high-dose radiation therapy as planned out of all patients starting this treatment and where the histological diagnosis is glioblastoma	≥90
VII	Proportion of patients finishing concomitant chemoradiotherapy with temozolomide as planned out of all patients starting this treatment and where the histological diagnosis is glioblastoma	≥85

### Patient completeness

Each Danish citizen is identified by a 10-digit code including information about sex and date of birth, which is registered in the Danish Civil Registration System [4]. This unique personal identification number is recorded in all public registries including the health service registries. The National Health Service provides regulated free access to health care services facilitating a population-based identification of all brain tumor patients in Denmark. To obtain mortality data, the DNOR-patients are linked to the Danish Civil Registration System on a monthly basis. The Danish Civil Registration System comprises daily updated data on the patients’ vital status, as well as date of death, disappearance, or emigration, if relevant [5].

The Danish National Patient Registry (NPR) [6] contains information about the dates of admission and discharge, the type of operation, examination, and treatment, as well as the main diagnosis and up to 20 sub-diagnoses for each discharge. The diagnoses are classified according to the ICD-10, and the surgical procedures according to the Danish classification system of surgical procedures [7]. The Danish National Pathology Registry (DPR) [8, 9] contains detailed nationwide records of all pathology specimens analyzed in Denmark since 1997.

To assess the patient completeness in the DNOR, recordings in both the NPR and the DPR were used as the gold standard. For each calendar year in the study period (2009–2014), the completeness was computed as the proportion of adult patients in the NPR and the DPR who were recorded in the DNOR, using the unique personal identification number to link between the registries.

For patients to be included in the gold standard population, three criteria all had to be fulfilled for each calendar year (2009–2014) (1) Age 18 years and older, (2) Registered in the NPR with one of the following ICD-10 codes: D33.0–33.2; D33.7; D33.9; D43.0–43.2; D43.7 + 9; C71.0–71.9; C72.8–9, and (3) Registered in the DPR with one of the following SNOMED M codes: M93813, M93823, M93841, M93853, M94003, M94013, M94113, M94213, M94243, M94253, M94403, M94423, M94503, or M94513.

### Validation of clinical variables

We compared the registration of selected variables between the DNOR and the medical records. A number of 100 patients with neurosurgery performed from 1 January 2013 through 31 December 2013 were randomly sampled from the DNOR. The variables were selected as being important according to the preoperative symptoms, diagnostic work-up, the surgical procedures, and the postoperative treatment (selected variables are listed in Table 3).

Data from the medical records of the 100 patients were extracted, collected, and keyed into a validation database by the same person, who was blinded to the original DNOR data. The data of the MRI characteristics was captured from what was written in the radiology report, and if missing then from what the neurosurgeon had written in the medical report. We did not reevaluate the pictures of the MRI if the description of the characteristics was missing in the radiology and medical report.

### Statistics

The validity was assessed as percent agreement between the originally reported data in the DNOR and data in medical records, including exact 95 % confidence intervals (CI). The chance-corrected proportional agreement was calculated as a kappa coefficient (k) for categorical variables. The following qualitative terms were attached to the kappa value: poor agreement if k ≤ 0.4, fair to good agreement if 0.4 < k < 0.75, and excellent agreement if k ≥ 0.75 [10].

### Approvals

The study was approved by the Danish Data Protection Agency (no. 2008-58-0035/14/6151) and by the Danish Health and Medicine Authority (no. 3-3013-514/1). According to Danish law no approval from the Ethics Board or patient’s consent is required as the study did not require intervention with the patients.

## Results

By 31 December 2014, a total of 2241 patients were recorded in the DNOR. The departments of neurosurgery only have access to open the data forms on primary diagnosis and surgery. Departments of oncology only have access to data forms on radiotherapy and chemotherapy. For 425 patients, only primary diagnosis and/or surgery were reported, 1606 patients had primary diagnosis, neurosurgical and oncological forms, and 210 patients had only radio- and chemotherapy forms, but no information on the primary diagnosis.

### Patient completeness in the DNOR

In 2009, 308 of 393 patients in the NPR/DPR (78 %) were present in the DNOR, whereas a higher completeness (93–97 %) was seen thereafter (Table 2). In the total study period, 2241 of 2437 patients (92 %) in NPR/DPR were registered in the DNOR.

**Table 2:** The patient registration completeness in the Danish Neuro-Oncology Registry (DNOR) using the Danish National Patient Register (NPR) and the Danish Pathology Registry (DPR) as reference

Year of diagnosis	NPR/DPR^a^	NPR/DPR –DNOR	DNOR	DNOR completeness
2009	393	85	308	78
2010	414	31	383	93
2011	395	26	369	93
2012	394	21	373	95
2013	413	14	399	97
2014	428	19	409	96
Total	2437	196	2241	92

^a^All patients registered in DNOR were also registered in NPR/DPR

### Validation of clinical DNOR variables

The medical records were available for all 100 patients and the observed agreements, and agreements corrected for chance agreement (kappa), are shown in Table 3.

**Table 3:** Agreement between clinical variables in the DNOR and the same information from the medical records estimated from a random sample of 100 patients

Selected clinical variables	Medical records/DNOR^a^ (numbers)	Agreement (%)	95 % CI (%)	Kappa^b^ (k value)
Onset symptoms				
Focal deficit	83/99	84	75–90	0.61
Seizure	89/99	90	82–95	0.71
Cognitive change	75/99	76	66–84	0.52
Headache	83/99	84	75–90	0.61
Diagnostic work-up				
Preoperative MRI performed	100/100	100	–	1.0
Date of preoperative MRI (±1 day)	83/99	84	75–90	–
Preoperative MRI: number of tumors	92/99	93	86–97	0.75
Preoperative MRI: contrast enhancement	85/87	98	92–100	0.49
Preoperative MRI: crossing midline	82/90	91	83–96	0.62
Preoperative MRI: localization	92/99	93	86–97	0.75
Preop. MRI: tumor diameter (±5 mm)	51/91	56	45–66	–
Preop. MRI: orthogonal diam. (±5 mm)	43/75	57	45–69	–
Surgery				
Procedure code of surgery	77/100	77	68–85	0.63
Date of surgery (±1 day)	99/100	99	95–100	–
Postoperative MRI performed	93/100	93	86–97	0.72
Date of postoperative MRI (±1 day)	76/82	93	84–97	–
Residual tumor on postoperative MRI	65/81	80	70–88	0.52
Radiotherapy				
Performance status before radiotherapy	53/57	93	83–98	0.87
Date of 1st radiation therapy (±1 day)	88/100	88	80–94	–
Treatment status, radiotherapy	95/100	95	89–98	0.43
Chemotherapy				
Performance status before chemotherapy	52/57	91	80–97	0.84
Date of 1st Chemotherapy (±1 day)	80/92	87	78–93	–
Treatment status, chemotherapy	82/91	90	82–95	0.63

*MRI* magnetic resonance imaging, *DNOR* Danish Neuro-Oncology Registry, *CI* confidence intervals

^a^Totals in DNOR varies between variables because not all information was available

^b^Kappa: poor agreement if k ≤ 0.4, fair to good agreement if 0.4 < k<0.75 and excellent agreement if k ≥ 0.75

#### Onset symptoms

Debut of focal neurological symptoms, epileptic seizures, changes in personality or behavior and cognitive deterioration, and headache may be present either mono-symptomatically or in combination. These symptoms at onset showed satisfying observed agreements on 84 % for focal deficit, 90 % for seizure, 76 % for cognitive change, and 84 % for headache. We found fair to good chance-corrected agreements, and especially high for epileptic seizures (k = 0.71).

#### Diagnostic work-up

The characteristics of the preoperative MRI are used to examine the surgical options. Registration of performed preoperative MRI showed excellent agreement (100 %; k 1.0). MRI characteristics had high agreements (91–99 %), with fair to good chance-corrected agreements (k 0.49–0.75). The agreement proportion for the scanning date was 84 % and for the bi-dimensional tumor diameter 56–57 %.

#### Surgery

The primary surgical procedure is planned to obtain a histological diagnosis and to remove as much tumor tissue as feasible, evaluated by an early postoperative MRI. The dates of operative procedure and postoperative MRI showed high agreements (93–99 %). Satisfying agreements were obtained for the surgical procedure code (77 %), the performed postoperative MRI (93 %) and residual tumor classification (80 %), which all had fair to good chance-corrected agreements (kappa 0.52–0.72).

#### Oncology

Residual tumor cells can be treated with radiochemotherapy in fit patients evaluated by the performance status score. Performance status before radio- or chemotherapy showed high agreements (91–93 %) with an excellent chance corrected agreement (k 0.84–0.87). Treatment status had fair to good chance-corrected agreements (k 0.43–0.63). The agreements were high for the date of onset of radio- or chemotherapy, 87–88 %.

## Discussion

The DNOR is a national population-based registry in which prospectively recorded clinical data from glioma patients in Denmark are registered. The results of this study show that the patient completeness of the database and the validity of clinical data are satisfactory. The completeness of the patient registration in the database was high and increased by calendar year, from 78 % in the 1st year to 96 % in 2014. Although reporting to clinical databases is mandatory by law, a low completeness of new clinical databases is not surprising, and is often related to lack of tradition for registration within the clinical disease area. From the start of the DNOR it has been ongoing work to motivate the clinical departments to increase the completeness. In the beginning, paper forms were sent to the departments for registration of data when evaluating the patient, and lists of potential missing patients were sent to the departments for review. In 2009, a total of 11 departments reported data, but as the treatment has been further centralized there are now only eight departments reporting data to the DNOR. Today, all the relevant departments have well-established online registration of data.

Exact evaluation of the completeness of clinical quality databases is often not possible due to lack of gold standard data. In Denmark, the unique personal registration number, given to all Danish residents, makes it possible to link individual patients in clinical quality databases to other registries from which a gold standard population can be determined. The completeness of the public health registries is high, partly due to the free access to treatment for all inhabitants and partly because hospitals only obtain public reimbursements for treatment if they ensure registration in the NPR. We used a combination of the NPR [6, 11] and the DPR [8]. The entire group of primarily operated glioma patients is expected to be registered in the NPR and DPR since the neurosurgical procedures are centralized in a few public university hospitals in Denmark. On the other hand, the general population of glioma patients may be larger because we have not included patients without a surgical procedure; i.e. not registered with a SNOMED M-code. Two Danish regional population-based studies showed that 19 % of the high-grade glioma patients with a dismal prognosis and 11 % of the low-grade glioma patients with a very good prognosis did not undergo surgery [12, 13].

The validity of key variables from DNOR was high. We selected the variables of major importance for the medical record review. A random sample of patients from all participating departments was identified. To standardize the data collection for study of validity we used the same assessor to monitor all the medical records from the 100 patients. This assessor had large experience in monitoring medical record data from clinical trials and was blinded to the original DNOR data.

Data on onset symptoms, diagnostic work-up, surgery and oncology treatment showed a good to excellent agreement. Tumor diameter from the preoperative diagnostic MRI had a lower agreement (56–57 %), which probably was because the surgeon often made his own evaluation of images giving a measure different from the radiology report. Thus, the disagreement may be due to interobserver variation and thus explained by the fact that we used the measures from the radiology report in this validation study and, only if data were missing here, did we use the measures from the neurosurgery text of the medical report.

DNOR uses the Eastern Cooperative Oncology Group performance status score [14], which is simpler than the original Karnofsky performance status [15], although they are believed to be interchangeable [16]. Performance status is an important prognostic factor in patients with glioma, and is used in the clinical decision process. The score is important, regardless of possible problems with the interobserver variability of the performance status score, which in other cancer studies has been rated moderate to high [16–18]. Pre-operative performance status is reported for almost every patient in the DNOR. However, none of the one hundred patients in the validity sample had a registration of a score for the pre-operative performance status in the medical report. Therefore, it was not possible to estimate the validity of the pre-operative performance status. Often, a description of the patient performance, but not a specific score, could be identified in the medical record. In the future it is recommended that the performance status score should be included also in the neurosurgical medical report. The postoperative performance status score before radiotherapy and chemotherapy showed excellent agreements.

Our study has a number of strengths. Registration in the DNOR is mandatory by law. The analyses were made at the individual level linked by the Danish national personal identification number. According to our knowledge, it is not possible to compare the validity of the selected variables in the DNOR to those in other brain tumor registries since such data on agreements have never been published before. However, high agreements on clinical variables have also been reported for other diseases in Danish clinical quality databases [19, 20]. Other national brain tumor registries, such as the Central Brain Tumor Registry of the United States [21] or the Austrian Brain Tumour Registry [22], have also reported high completeness of patients, although this has not been verified by comparing to a gold standard population as in this study. Regional brain tumor registries in France [23] and Sweden [24] have also reported high completeness.

The patient completeness of the DNOR database and the validity of registered data are of crucial importance when using clinical quality databases as a research tool [25]. This study shows that DNOR has high patient registration completeness and a good validity of clinically important variables, making it a robust research tool. However, to further increase the completeness of DNOR there is ongoing work to make advantage of data from the NPR and the DPR by implementing generic algorithms from the Danish National Clinical Cancer Database workgroup. This workgroup under the Danish Clinical Quality Improvement Program has described procedures for automated retrieval of data from the NPR and the DPR. In an automated process the centrally retrieved data are interpreted by disease specific algorithms and sent to the clinicians for review to assure validation and supplementation of clinical data in the DNOR.

In conclusion, the completeness of the patient registration in the DNOR and the validity of clinical patient data was high. The DNOR is a well-established database, it is a reliable source for future scientific studies, and it will be used for ongoing clinical quality assessments among patients with gliomas.

## Abbreviations

CI: confidence intervals
DNOG: Danish Neuro-Oncology Group
DNOR: Danish Neuro-Oncology Registry
DPR: Danish National Pathology Registry
ICD-10: international classification of diseases tenth edition
k: kappa
MRI: magnetic resonance imaging
NCR: National Cancer Registry
NPR: Danish National Patient Registry
SNOMED: systematized nomenclature of medicine
